# Supplementary material for: Direct administration of the non-competitive interleukin-1 receptor antagonist rytvela transiently reduced intrauterine inflammation in an extremely preterm sheep model of chorioamnionitis
Source: PLoS One. 2021 Sep 24;16(9):e0257847. doi: 10.1371/journal.pone.0257847 (PMC8462743; doi:10.1371/journal.pone.0257847)

GFAP Total Protein Target band

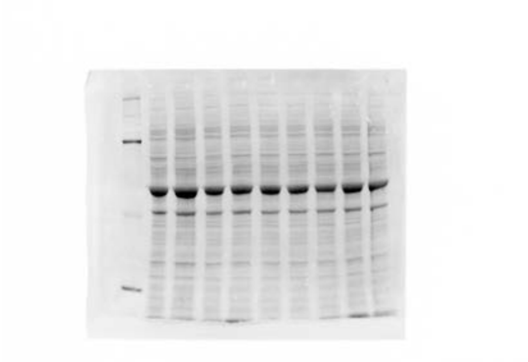

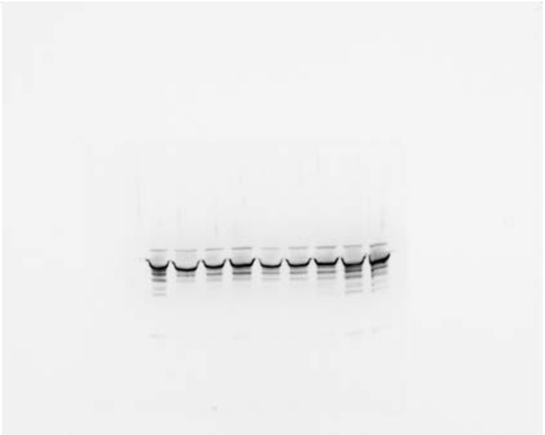

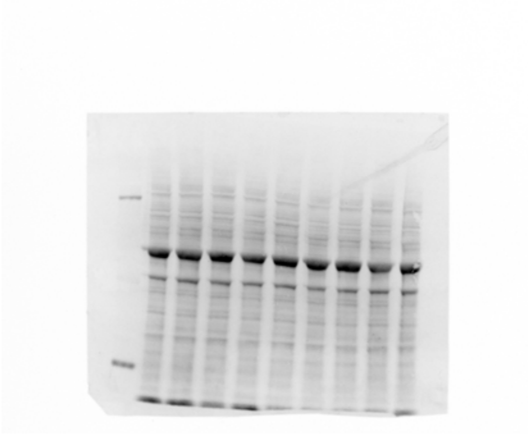

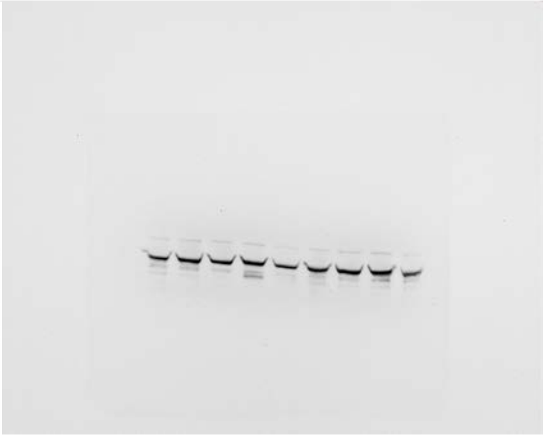

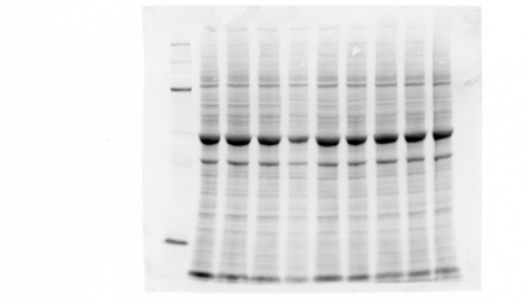

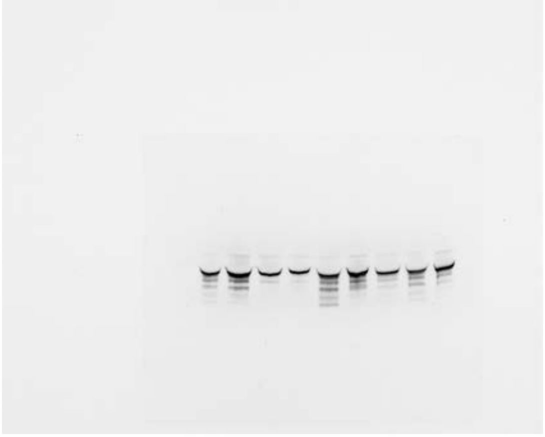

IBA-1 Total Protein Target band

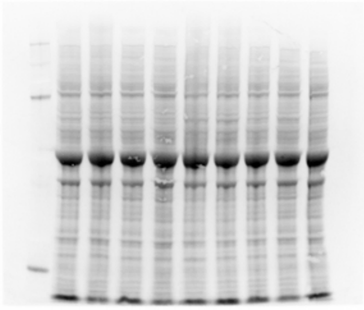

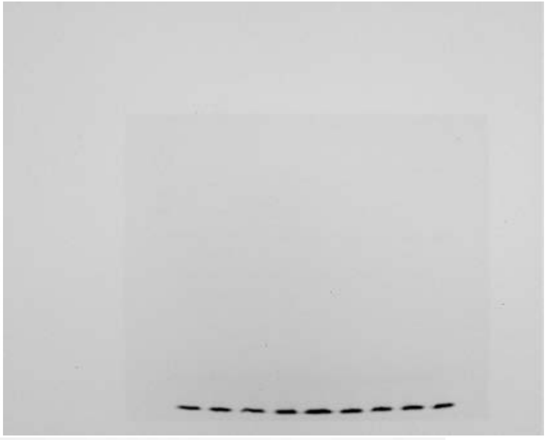

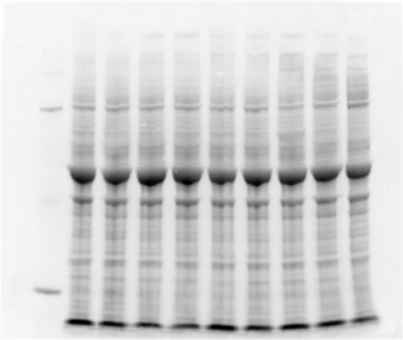

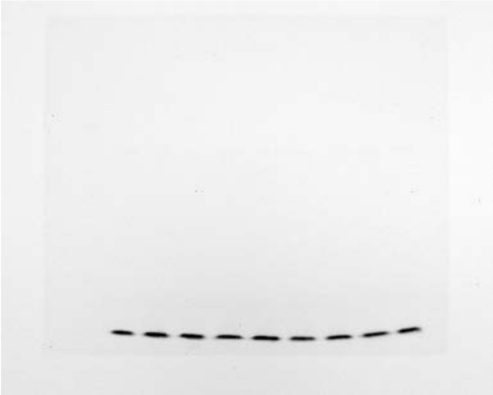

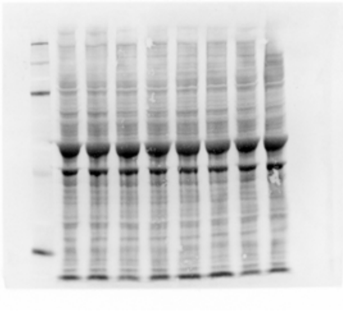

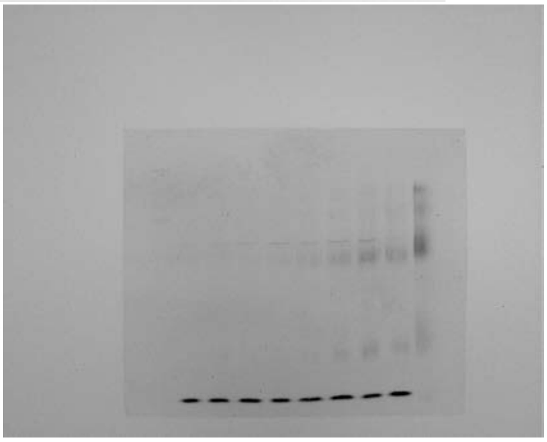

Olig-2 Total Protein Target band

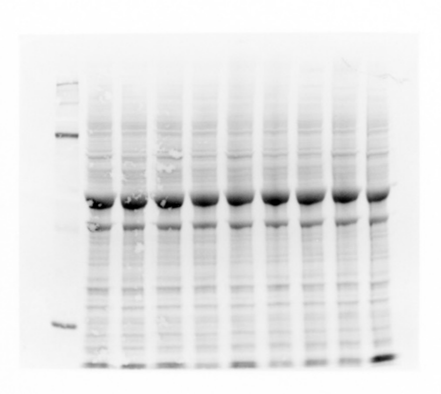

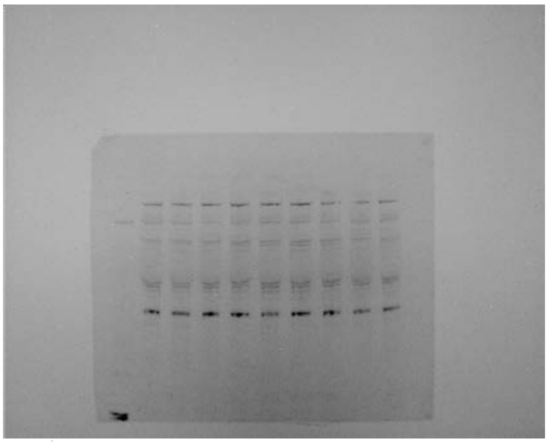

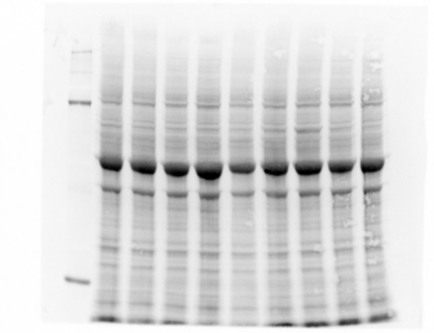

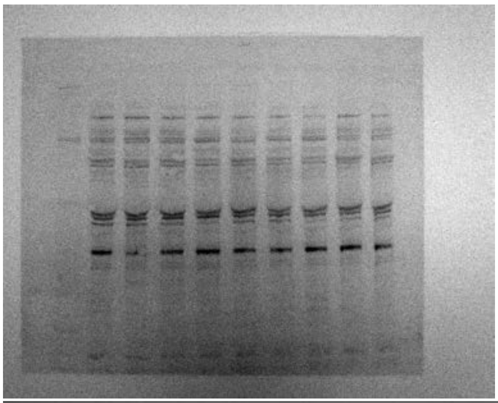

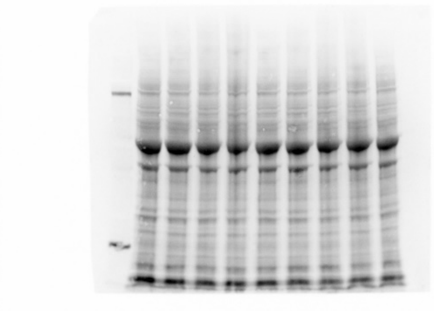

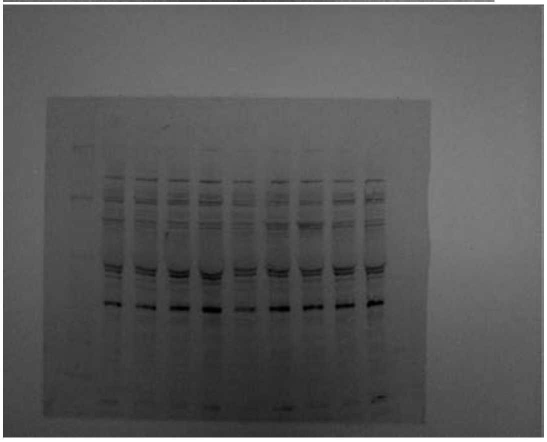

NeuN Total Protein Target band

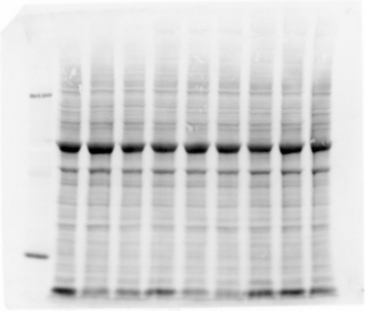

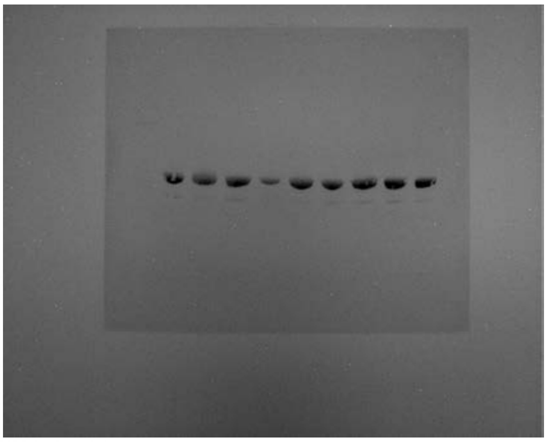

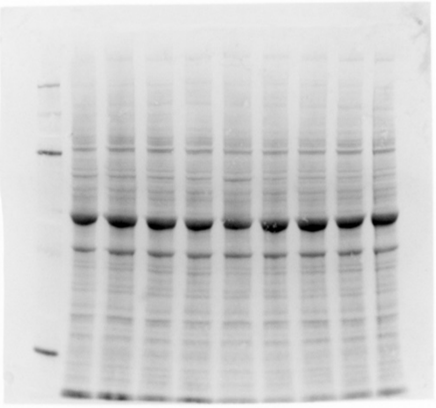

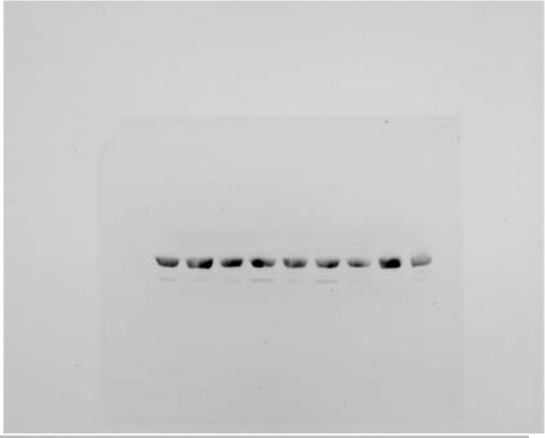

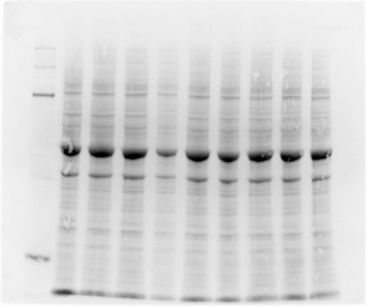

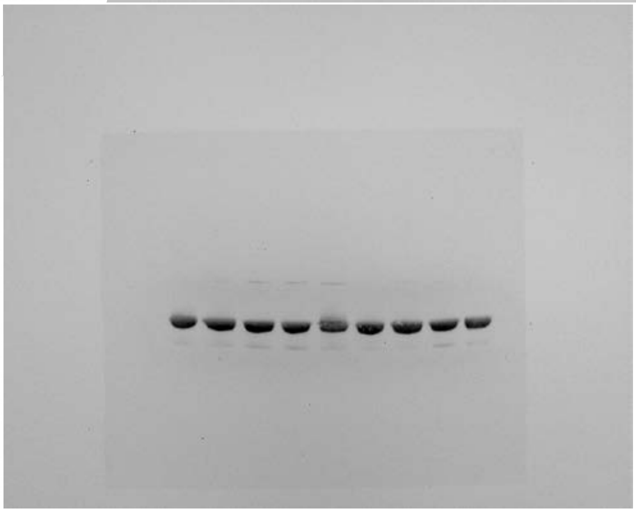

Supplement: S1 Raw images — (PDF) [file pone.0257847.s001.pdf]
