# Supplementary material for: Direct administration of the non-competitive interleukin-1 receptor antagonist rytvela transiently reduced intrauterine inflammation in an extremely preterm sheep model of chorioamnionitis
Source: PLoS One. 2021 Sep 24;16(9):e0257847. doi: 10.1371/journal.pone.0257847 (PMC8462743; doi:10.1371/journal.pone.0257847)
Supplement: Attachment — Submitted filename: PLOS Response to Reviewers 18.06.21.docx [file pone.0257847.s002.docx]

Journal requirement amendments:

1) ORCID

Now linked to an ORCID ID

2) Reference list

OK

3) Western Images

Included in supplemental file

4) Reference to ‘data not shown’

Removed from manuscript. All relevant data now contained within body of manuscript.

Review Responses:

On behalf of the author team I would like to thank the reviewers for their positive assessment of our work. We have responded to queries in point form, as below, and identified changes in the manuscript (please also see track changes in the uploaded manuscript).

Reviewer #1:

**R1C1: *Please cite the literature that the used rytvela concentration is based on.***

We have included references to the papers from which the dose of rytvela used in this study was derived.
Line 147-148

**R1C2: *Please explain the lack of an inflammatory response to LPS concerning TNF-a, IL-8 and IL-1b levels.***

The Reviewer raises a very important point; the half-life time and dynamics of the cytokines/chemokines are different depending on fetal organs or amniotic fluid. The expression of RNA transcripts for IL-1β in lung, skin and colon and TNF-α in fetal skin and chorioamnion were significantly increased in LPS Group compared with Saline Group. Additionally, histological assessment of fetal lung and chorioamnion in the LPS Group also showed that an inflammatory response had occurred in response to LPS exposure.

With regards the lack of a significant difference in amniotic fluid inflammation, it is possible that the effect of surgery and the production of AF cytokines over the 48h recovery period increased the experimental noise to a level sufficient to mask the LPS-driven inflammatory response. We have included reference to these potential factors in the revised manuscript.

Line 456-457, 472-474

**R1C3: *As Fig. 3 displays a pre-intervention analysis, no results for rytvela are shown, so please remove rytvela from the text and figure, as this is confusing.***

Apologies for not clearly articulating the Figure 3. We have changed “the LPS Group and the LPS + rytvela Group” to “LPS-treated Groups”. We have combined Figure 2 and Figure 3 as the Reviewer#2 mentioned. Therefore Figure 2-B is the now previous Figure 3.Line 284-286, 288-290, 299, 302-304 and Figure 2-A, Figure 2-B

**R1C4: *Line 258: please correct Mann-Whitney test***

Thank you for pointing this out – error now corrected.

Line 259

**R1C5: *In the discussion the advantages of rytvela compared to other IL-1b antagonists should be discussed, as this is an important aspect of consideration for a future clinical translation. Ideally rytvela should be directly compared to a common IL-1b antagonist, but the reviewer understands that this is out of scope for the current study. A comment in the discussion would be appreciated.***

We agree that this is a useful addition to the study. Unlike other IL-1β antagonists, rytvela exerts functional selectivity and doesn’t inhibit NF-κB activation. Considering that previous studies have suggested complete inhibition of NF-κB activation may be undesirable for pregnancy, this potential advantage of rytvela has been highlighted as it might be an important feature for any future clinical translation.

Line511-516

**Reviewer #2:**

**R2C1: *What was the rationale of the dose of LPS used? Can the authors cite prior publications that have used a similar dose?***

We have previously used a 10 mg dose LPS to the amniotic cavity in the preterm sheep model, which reliably induces chorioamnionitis and a fetal inflammatory response. We have reference to several previous publications using this LPS dose.

Line90-92

**R2C2: *Please include the number of biological replicates in all figure legends and represent the individual samples on the plots.***

We have amended figure legends as suggested.

**R2C3: *What is the clinical relevance of inflammation on the skin surface as this is a main finding of the manuscript?***

The fetal skin is the one of the first tissues to be exposed to microbial stimulation following infection/colonization of the amniotic fluid into fetal body. Although the skin inflammation may not cause localized damage (as is seen with lung or skin) it may have the ability to generate a systemic inflammatory signal given its large percentage of total fetal weight and extensive vascular supply. As this study was designed to explore the presence of fetal tissue inflammation in the setting of LPS and rytvela treatment is important asses fetal skin inflammation. We have updated the revised manuscript accordingly to better reflect this.

Line491-493

**R2C4: *In Figure 4, TNF-alpha had additional time-points at 36- and 60-hours while the other mediators do not.***

Thank you for pointing this out. We have standardized the time points between figures.

Figure 3

**R2C5: *Figure 8 legend also has description for results from Figure 9A*.**

Line 389-392 is part of the main manuscript, and is not part of the legend for Figure 8. Apologies if this was not clear.

**R2C6: *Can the authors clarify the relation between IL1-beta and MCP-1 expression? Since the AF did not show any changes in IL-beta but did show a decrease in MCP-1, this is worthy of further clarification. Does it have off-target effects?***

MCP-1 is a downstream product of IL-1 β signaling. rytvela selectively inhibits IL-1Racp. Thus, because rytvela doesn’t inhibit or extinguish IL-1β signaling directly, the significant decreases of MCP-1 in AF of the LPS + rytvela Group suggest that rytvela is acting to inhibit, at least in part, downstream of the IL-1 activation.

Considering the IL-1β concentrations of fetal lung, LPS animals showed significant increases compared with Saline Group, while there was no differences between the LPS + rytvela Group and the Saline Group. IL-1β mRNA expression in the skin of fetuses in the LPS + rytvela Group was significantly lower than that of animals from the LPS Group. As such, our data show that rytvela reduced MCP-1 in the fetal skin but not in the amniotic fluid.

Taken together, the significant decreases of MCP-1 expression in AF suggested a partial inhibition of inflammation induced by rytvela rather than off-target effects.
As for the results of the post-intervention analysis (Figure 3), we believe that the combined effects of a limited number of animals and time-course samples combined with experimental noise post-surgery impacted our ability to identify potential differences in amniotic fluid cytokine levels. In the future, it may be worthwhile to avoid a 48h recovery period in inflammatory studies so as to minimize the amount of surgery-induced background inflammation.

Line 541-543

**R2C7: *What was the rationale behind the neuronal markers studied?***

A fetal inflammatory response mediated by cytokine/chemokine expression leads to brain damage in both human fetus, and in animal inflammation models when exposed to LPS. Reactions of the fetal brain to LPS inflammation include: 1) activated microglia, 2) activated astrocytes, 3) maturation arrest or death of oligodendrocytes, and 4) axonal loss or death of neurons. Therefore we chose to assess cellular changes in the brain using the following markers; GFAP for astrocytes, IBA-1 for microglia, Olig-2 for oligodendrocytes and NeuN for neurons, respectively.

Line392-394

**R2C8: *Figures 2 and 3 can be combined***

We agree and have amended accordingly.

Figure 2-A, B

**R2C9: *Was any staining used specifically for the identification of the inflammatory cells?***

No, we didn’t conduct other staining to look for specific cell populations. This will be an important part of future studies using this agent.

N/A
